# Clonal analysis and virulent traits of pathogenic extraintestinal *Escherichia coli* isolates from swine in China

**DOI:** 10.1186/1746-6148-8-140

**Published:** 2012-08-21

**Authors:** Yi Ding, Xibiao Tang, Ping Lu, Bin Wu, Zhuofei Xu, Wugang Liu, Ruixuan Zhang, Weicheng Bei, Huanchun Chen, Chen Tan

**Affiliations:** 1 State Key Laboratory of Agricultural Microbiology, Huazhong Agricultural University, Wuhan, 430070, People’s Republic of China; 2 College of Veterinary Medicine, Huazhong Agricultural University, Wuhan, 430070, People’s Republic of China; 3 China Animal Health and Epidemiology Center, Qingdao, 266032, People’s Republic of China

**Keywords:** Extraintestinal *Escherichia coli*, MLST, Virulence gene, Pig

## Abstract

**Background:**

Extraintestinal pathogenic *Escherichia coli* (ExPEC) can cause a variety of infections outside the gastrointestinal tract in humans and animals. Infections due to swine ExPECs have been occurring with increasing frequency in China. These ExPECs may now be considered a new food-borne pathogen that causes cross-infections between humans and pigs. Knowledge of the clonal structure and virulence genes is needed as a framework to improve the understanding of phylogenetic traits of porcine ExPECs.

**Results:**

Multilocus sequence typing (MLST) data showed that the isolates investigated in this study could be placed into four main clonal complexes, designated as CC10, CC1687, CC88 and CC58. Strains within CC10 were classified as phylogroup A, and these accounted for most of our porcine ExPEC isolates. Isolates in the CC1687 clonal complex, formed by new sequence types (STs), was classified as phylogroup D, with CC88 isolates considered as B2 and CC58 isolates as B1. Porcine ExPECs in these four clonal complexes demonstrated significantly different virulence gene patterns. A few porcine ExPECs were indentified in phylogroup B2, the phylogroup in which human ExPECs mainly exist. However some STs in the four clonal groups of porcine ExPECs were reported to cause extraintestinal infections in human, based on data in the MLST database.

**Conclusion:**

Porcine ExPECs have different virulence gene patterns for different clonal complexes. However, these strains are mostly fell in phylogenentic phylogroup A, B1 and D, which is different from human ExPECs that concentrate in phylogroup B2. Our findings provide a better understanding relating to the clonal structure of ExPECs in diseased pigs and indicate a need to re-evaluate their contribution to human ExPEC diseases.

## Background

*Escherichia coli* (*E. coli*) are common bacteria that colonize the intestinal tract as normal flora and are known to cause a variety of diseases. Extraintestinal pathogenic *E. coli* (ExPEC) is epidemiologically and genetically different from intestinal pathogenic or commensal *E. coli.* A typical ExPEC does not cause enteric diseases; however, it can asymptomatically colonize the intestinal tract*,* or cause extraintestinal diseases in both normal and compromised hosts [1,2]. The major virulence factors present in ExPECs are distinct from those found in other *E. coli* strains; ExPECs possess a variety of the combination of virulence-associated genes instead of a common virulence mechanism to upset the host defense system [3]. ExPECs of human origin were defined by Johnson et al. [4] as *E. coli* isolates containing two or more virulence markers: *papA/papC, sfa/foc, afa/dra, kpsMTII* and *iutA*. The same criterion was also used to define ExPECs isolated from other animal species because fewer systematic studies were carried out on ExPEC from these animals with respect to virulence genes.

Within the Chinese pig industry, porcine ExPEC diseases have become more prevalent. This has corresponded to an increase in the number an increase in the number of resistant bacteria and immunocompromised diseases [5]. The frequency of isolation of ExPECs from diseased pigs increased from 3.1% in 2004 to 14.6% in 2007. Moreover, strains possessing similar virulence profiles and serogroups to human ExPECs have been detected from our collection of porcine ExPECs, suggesting that these pathogens may have strong zoonotic potential [6]. The virulence and zoonotic potential of porcine ExPECs may be linked to a specific clonal lineage within phylogenetic groups. It is important to understand the population structure of porcine ExPECs. However, the relevant information is rarely available.

Multilocus sequence typing (MLST) is a powerful method that can be applied to comparing genotypes and global surveillance of pathogens. MLST is based on the sequencing of 6–7 selected housekeeping genes and identification of polymorphic nucleotide sites. A combination of the alleles at the 6–7 loci provides a unique sequence types (ST) identifier that can be used to discriminate strains. A new implementation of algorithm, eBURST, has been shown to be effective in the analysis of MLST data. Related sample strains are grouped into a clonal complex (CC), and the STs which demonstrated the greatest number of single-locus variants (SLVs) were assigned as the founder. Compared with a dendrogram, eBURST provides more information about founder genotypes and patterns of the evolutionary descent.

In the present study, a collection of 81 ExPEC strains isolated from diseased pigs within China from 2006–2007 were subjected to MLST, analyzed via serogrouping and by virulence gene typing with the aim of improving our understanding of the clonal structure and virulence traits of swine ExPECs.

## Results

### MLST and eBURST analysis of porcine extraintestinal *E.coli*

The CCs and the distributions of serogroups and virulence genes are shown in Table 1. Seven housekeeping genes of all 81 porcine ExPEC strains were successfully amplified and sequenced. A total of 43 STs was found, of which 29 were reported previously. Fourteen STs from 30 strains were identified as new STs and designated ST1687, 1688, 1690, 1691, 1692, 1694, 1696–1702, and 1731 ( http://mlst.ucc.ie/mlst/dbs/Ecoli/GetTableInfo_html).

**Table 1:** Distribution of serogroups and virulence genes in clonal complexes

**Serogroups and virulence genes**		**Distribution of serogroup and virulence gene within CCs, no. (%)**			
		**CC10 (n = 26)**	**CC58 (n = 8)**	**CC1687 (n = 19)**	**CC88 (n = 6)**
Serogroup^b^	O11	3/(11.1)	0/(0)	14/(73.7)^c^	0/(0)
	O8	6/(22.2)	3/(37.5)	0/(0)	3/(15.3)
	O161	1/(3.7)	0/(0)	1/(5.3)	1/(5.3)
	O138	0/(0)	2/(25.0)	0/(0)	1/(5.3)
Virulence gene^a^	O101	5/(18.5)	0/(0)	0/(0)	0/(0)
	*papA*	23/(88.5)	5/(62.5)	14/(73.7)	3/(50.0)
	*papC*	10/(38.5)	3/(37.5)	6/(31.6)	4/(66.7)
	*sfa*	20/(76.9)	7/(87.5)	5/(26.3)^c^	2/(33.3)
	*Afa/draBC*	9/(34.6)	2/(25.0)	2/(10.5)	1/(16.7)
	*kpsMTII*	21/(80.8)	6/(75.0)	18/(94.7)	4/(66.7)
	*iutA*	21/(80.8)	6/(37.5)	16/(84.2)	4/(66.7)
	*papEF*	2/(7.7)	0/(0)	1/(5.3)	2/(33.3)
	*papG allele I*	1/(3.8)	0/(0)	1/(5.3)	0/(0)
	*papG allele II*	0/(0)	0/(0)	0/(0)	2/(33.3)
	*papG allele III*	0/(0)	0/(0)	0/(0)	0/(0)
	*papG allele II/III*	0/(0)	0/(0)	0/(0)	2/(33.3)
	*sfaS*	3/(11.5)	0/(0)	1/(5.3)	2/(33.3)
	*focG*	1/(3.8)	1/(12.5)	1/(5.3)	0/(0)
	*bmaE*	1/(3.8)	0/(0)	5/(26.3)	0/(0)
	*gafD*	3/(11.5)	0/(0)	0/(0)	1/(16.7)
	*nfaE*	5/(18.5)	2/(25.0)	4/(21.1)	0/(0)
	*iha*	1/(3.8)	0/(0)	1/(5.3)	1/(16.7)
	*fimH*	23/(85.2)	8/(100)	19/(100)	6/(100)
	*cdtB*	0/(0)	0/(0)	1/(5.3)	0/(0)
	*cvaC*	17/(63.0)	6/(75.0)	19/(100)	6/(100)
	*hlyA*	2/(7.6)	0/(0)	4/(21.1)	1/(16.7)
	*CNF1*	5/(18.5)	4/(50.0)	1/(5.3)	0/(0)
	*kpsMT III*	2/(7.6)	0/(0)	1/(5.3)	0/(0)
	*kpsMT K1*	5/(18.5)	1/(12.5)	16/(84.2)^c^	1/(16.7)
	*kpsMT “K5”*	7/(26.9)	2/(25.0)	16/(84.2)	2/(33.3)
	*fyuA*	12/(44.4)	5/(62.5)	12/(63.2)	4/(66.7)
	*ireA*	19/(70.4)	7/(87.5)	12/(63.2)	5/(83.3)
	*Irp2*	13/(50.0)	7/(87.5)	12/(63.2)	6/(100)
	*iron*	16/(59.3)	6/(75.0)	17/(89.5)	6/(100)
	*ibeA*	15/(55.6)	6/(75.0)	1/(5.2)^c^	5/(83.3)
	*ompT*	4/(15.4)	6/(75.0)^c^	2/(10.5)	3/(50)
	*traT*	25/(96.1)	8/(100)	18/(94.7)	6/(100)
	*iss*	10/(38.4) ^c^	7/(87.75)	16/(84.2)	6/(100)
	*tsh*	3/(11.5)	0/(0)	0/(0)	3/(50)
	PAI	2/(7.6)	0/(0)	1/(5.3)	1/(16.7)

^a^All genes were detected by PCR.

^b^Serogroup was determined using antisera from China Institute of Veterinary Drug Control (Beijing, China).

^c^*P* < 0.01, indicated group versus all other groups combined.

**Figure 1:**
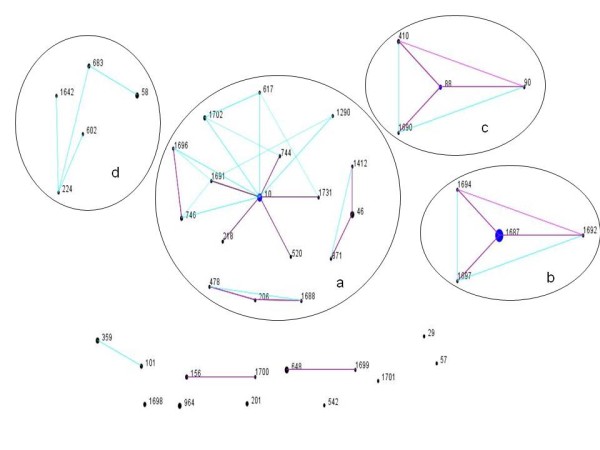
**eBURST analysis of porcine extraintestinal *Escherichia coli.*** Clonal structure of ExPEC is demonstrated by eBURST plot based on the profile of seven allelic housekeeping genes. Strains share five of seven alleles was grouped into a clonal complex. Porcine pathogenic ExPEC strains comprised four main CCs (**a**: clonal complex 10, **b**: clonal complex 1687, **c**: clonal complex 88, and **d**: clonal complex 58), two doublets and 9 singletons. *E. coli* strains from the same ancestor based on the whole MLST data were grouped together with black circles. The ancestral ST is denoted in blue and the derived and ungrouped STs are in black. Individual strains responsible for SLVs and DLVs among these CCs are connected to their ancestral ST with the black and purple lines, respectively.

The two most frequent STs were ST1687 (19.8%) and ST10 (7.4%). eBURST analysis of 81 strains showed that there were five CCs, CC10, CC1687, CC88, CC46 and CC206, and two doublets. Nine isolates, including two of the newly identified STs (ST1698 and ST1701), were defined as a singletons. Further comparison of the porcine ExPECs with the entire MLST database indicated that CC46, CC206 and several singletons were derivatives of CC10, and that five singletons (ST224, ST58, ST1642, ST602 and ST683) were related to strains from CC58 (Figure 1). These related STs and CCs, which did not fall into a single CC but were grouped together according to the MLST database, were classified as CC10 and CC58 respectively in this study (Table 1). The strains within CC10 and their derivatives consisted of 26 isolates (32.1%), in which ST10 and ST46 occurred most frequently and accounted for 38.5% (n = 10) of STs.

### Phylogenetic grouping

E. coli can be classified as phylogroup A, B1, B2 or D according to the phylogenetic relationship of the sequences. Twenty-six isolates belonged to phylogroup A, 20 to phylogroup B1, 10 to phylogroup B2, and 25 to phylogroup D. The isolates belonging to phylogroups B2 and D accounted for 43.2% of all isolates. This was slightly lower than the proportion of the, 8isolates belonging to phylogroups A and B1 combined. The strains falling into phylogroup B2 accounted for 12.3% of all isolates. No significant correlation between serogroup and phylogenetic group was found, except that all isolates of serogroup O11 with ST1687, one of the newly identified STs, belonged to phylogenetic group D.

### Distribution of serogroups in CCs

No difference in distribution of the five most frequent serogroups (O11, O8, O138, O161, and O101) among CCs was observed, except that serogroup O101 belonged to CC10, and serogroup O11 was predominant in CC1687. In CC58, doublets and singletons from several serogroups were dispersed among isolates of the same CC or ST, indicating that surface antigens of the strains in these CCs experienced robust recombination.

### Association of CC with virulence genes

Association of the three major CCs with virulence genes was observed in our study. Both the *kpsMT K1* and *ompT* genes were positively associated with CC1687 and CC58, respectively. The *sfa* and *iss* genes were negatively associated with CC1687 and CC10, respectively.

### Geographical location and tissue origin of ExPECs

The eighty-one ExPECs were isolated from ten provinces in china, mainly from Hubei (n = 30) and Hunan (n = 29). The strains in CC1698 concentrate in Hunan province. Tissue origin of the samples varied, including lung, liver, spleen, pericardial sac, brain, blood, lymph, joint, and hydrops. The majority of the tissues from which ExPECs were collected were lung (46.9%), blood (12.3%) and pericardial sac (11.1%), The statistical analysis showed that no significant correlation was found between geographical location and tissue of origin.

## Discussion

Extraintestinal *E. coli* can lead to a variety of diseases outside the intestinal tract in a broad range of hosts. To date, most research has focused on the relationship between ExPECs in birds with those in human [7-9]. However, ExPECs of swine origin have not been well documented and investigated. The importance of swine ExPECs is not fully appreciated at present. This is mainly because the phylogenetic types present in swine differ considerably with the majority of ExPEC isolates in human, and because There have been much lower number of ExPECs isolated from pork compared with poultry meat [10,11].

Studies by Dezfulian et al. [10] and Marynard et al. [11] showed that greater than 50% of ExPECs from diseased pigs belonged to phylogenetic groups A or B1, whereas most ExPECs from human clinical cases belonged to phylogroups B2 and/or D. A survey of ExPECs in food products carried out by Johnson et al. [12] in Minneapolis–St. Paul retail establishment in 1999–2000 revealed that no ExPECs were isolated from pork. However, ExPECs from pork have been detected in subsequent reports. A survey by Johnson et al. [13] in the same area reported that ExPEC was isolated from b 18.9% of samples of raw beef and pork in 2001–2003. Xia et al. [14] reported in 2006 that ExPECs were isolated from 8.3% of pork chops in Georgia, Maryland, Oregon, and Tennessee. Data from our clinical microbiology laboratory also showed that porcine ExPEC diseases increased five times from 2004 to 2007 in the 19 province of China [6]. These investigations provide an important clue that the occurrence of ExPEC in pork has increased gradually over recent years. This increasing trend may be caused by the over- or misuse of antibiotics and/or bacteria diseases infecting immunocompromised pigs [15-18]. Multidrug resistance of porcine ExPEC strains and immunosuppressive diseases may promote the incidence of porcine ExPEC diseases in China. Therefore, swine ExPECs may comprise a significant proportion of ExPECs in the retail food sold throughout China, again highlighting the importance of why their molecular epidemiology must be better understood.

Most researchers have found that ExPECs of human and avian origin belong to phylogenetic group B2 and, to a lesser extent, to phylogroup D. Sporadically, these ExPECs have appeared in phylogroups A and B1. The majority of phylogroup A and B1 isolates are mainly the commensal and intestinal pathogenic strains. Moreno et al. [19] reported that ExPECs of phylogroups A and B1 presented low virulent potential and could cause sporadic diseases in compromised patients only. In our study, porcine ExPECs were mainly distributed among phylogroups A, B1 and D, but a few strains were classified as phylogroup B2. The discrepancy in phylogenetic types between swine ExPECs and human ExPECs could be explained by host specificity.

MLST analysis is a powerful phylogenetic tool with high reproducibility, thus facilitating global comparisons. By analyzing multiple housekeeping gene loci, strains with related phylogenetic background can be grouped and analyzed together [20]. Comparison of porcine ExPECs with the MLST database showed that most of the clones observed in our study possessed the same allele profiles as human ExPEC clones reported elsewhere, especially those from South America, North America and Europe. Isolates of ExPECs in CC10 were first detected in Sweden and later seen in other parts of Europe. These were found to caused urinary infections in humans ( http://mlst.ucc.ie/mlst/dbs/Ecoli/GetTableInfo_html). Moreno et al. [19] reported that ExPEC strains in this group caused extraintestinal infections in compromised patients. Some of the STs, such as ST520, ST218 and ST746 in CC10, were reported exclusively as diarrheagenic *E. coli* rather than ExPEC diseases. ExPEC strains in these clonal groups may be derived from intestinal pathogenic *E. coli* strains and have evolved into ExPEC through accumulation of virulence factors [21]. Additionally, the new ST1687 and its related strains, with homogeneous phylogenetic background and belonging to the same serogroup, are described here for the first time. The large number of the novel STs suggests that they may prevail in Hunan and possess advantageous traits that have evolved independently. The molecular and epidemiological characteristics of the ST1687 strains require further surveillance and investigation.

The correlation between CC and virulence factors was also investigated in this study. Significant differences were detected between the four main clonal groups with respect to patterns of virulence genes. Strains in CC10 had significantly fewer *iss* genes, while strains in CC58 had more *ompT* genes. The *iss* gene is a plasmid-associated gene that has been reported to be widely distributed among avian extraintestinal pathogenic *E. coli* (APEC) [22]. The *ompT* gene is a protease that cleaves colicin A E1 E2 E3. However, the role of *iss* and *ompT* in the pathogenesis of APECs or porcine ExPECs is unclear. Strains in CC1648 had few *sfa* and *ibeA* genes but more *kptM K1* gene. *Sfa* is a Dr-binding adhesion confirmed to be associated with E.coli causing neonatal meningitis and urinary infection. The *ibeA* gene and K1 gene are reported to be important in the pathogenesis of NMECs [23]. The presence or absence of these genes may be a trait inherited by clonal lineage and attributed to special pathogenic mechanisms. These results were consistent with those in a previous study on ExPECs from humans and other animals, in which ExPECs exhibited miscellaneous virulence gene patterns [21]. Porcine ExPECs in different CCs probably also infected pigs via different mechanisms as human ExPECs. We also observed that the ExPEC isolated from diseased pigs possessed different virulence gene patterns as those isolated from retail pork. Xia et al. [14] reported that afa/draBC was not detected in 200 ExPEC strains isolated from pork, but this gene was detected in the four main clonal groups of porcine ExPECs. Therefore, the source of ExPECs detected in retail pork may be due to contamination during processing, storage, or transportation.

The presence of ExPECs in food products suggests that ExPECs may represent a new class of foodborne pathogens. Although pork is a common meat source that is consumed by a large number of people, the zoonotic potential of porcine ExPECs has not been thoroughly investigated. Studies on ExPEC strains in pork or diseased pigs have been limited to a few areas and sample pools [9,10,24]. There is a need to expand such studies to more countries to obtain a better picture of the profile of porcine ExPECs and its link to human diseases. Our study provides preliminary data to understand the association of clonality with virulence gene traits in porcine ExPECs. We also find that the predominant serogroups of porcine ExPECs are serogroup O11, O8, O138, O161, O101, and that clonal complex 10 and clonal complex 1687 are the main porcine ExPEC clonal lineages in China. The contribution of Porcine ExPECs to human ExPEC diseases could be more convincing and better explained if there is more information about the clonality, phylogenetic group, and virulent trait of commensal *E. coli* and human ExPECs in China.

## Material and methods

### Strains isolation and characterization

ExPEC (n = 81) strains were isolated from the samples of lung, brain, blood, spleen, and Pericardial sac of pigs with septicemia, meningitis and respiratory disease by the Clinic Microbiology Laboratory of Huazhong Agricultural University from 2006–2007. These samples came from the southern and central provinces of China. Each *E. coli* from the samples was collected from different pigs and confirmed with a rapid identification system (Biolog MicroStation). Mutiplex PCR described by Johnson et al. [4] was used to amplify five virulence genes: *papA* and/or *papC* (P fimbriae); *sfa/foc* (S and F1C fimbriae); *afa/dra* (Dr-antigen-binding adhesins); *iutA* (aerobactin receptor); and *kpsMT II* (group 2 capsular polysaccharide units). *E. coli* that possessed at least two of the five virulence genes were thought to be ExPECs. Serogrouping of porcine ExPEC isolates was performed with an *E. coli* serogrouping kit (China Institute of Veterinary Drug Control, Beijing, China). Phylogenetic grouping was conducted using the method of Clermont et al. [25]. Triplex PCR was applied to detect *chuA, yjaA* and EspE4.C2 genes in swine ExPECs. Based on the presence or absence of these three genes, all ExPEC isolates were divided into four main phylogenetic groups according to the following criteria: isolates of *chuA*– and TspE4.C2– were assigned to group A; *chuA*–/TspE4.C2+ isolates were assigned to group B1. *chuA*+/*yjaA* + Isolates were assigned to group B2; *chuA*+/*yjaA*– isolates were assigned to group D. There are 35 different virulence genes that were thought to be associated with human and/or avian extraintestinal diseases (Table 1) that were assessed in this study based on reports from Johnson et al. [26], Chapman et al. [27] and Ewers et al. [28]. The amplified products were analyzed using 0.8% agarose gel electrophoresis, and recorded with a gel documentation system. Results for each virulence factor that conflicted initially were investigated further and sequenced for verification. The details of these procedures are described in our previous paper [6].

### MLST assay

Seven housekeeping genes (*adk, fumC, gyrB, icd, mdh, purA and recA*) were amplified uing primers and protocols outlined on the *E. coli* MLST website ( http://mlst.ucc.ie/mlst/dbs/Ecoli). The PCRs were carried out in a 50-μl mixture containing 1 μl of DNA, 1 μl of each primer, 5 μl of primer star buffer, 4 μl of dNTP mixture, 38.5 μl of deionized distilled water, and 0.5 μl of primer star DNA polymerase. The amplified PCR products were confirmed with 0.8% (w/v) agarose gels through electrophoresis, and then sequenced by Sangon Biological Engineering Technology Inc (Shanghai, China). The sequences were compared with those of the seven housekeeping genes contained in the *E. coli* MLST database to obtain allelic profiles and sequence type (ST).

### Statistical analysis

The proportion of distributions of the strains in clonal groups according to their serogroups and virulence genes were evaluated by Fisher’s exact test (two-tailed). Comparisons between different groups were tested, and if a significant difference was observed, comparisons between each group and all other groups combined were evaluated. A P-value less than 0.01 was considered significant. All statistical analyses were performed with SPSS version 17.0 (SPSS, Chicago, IL, USA).

## Competing interests

The authors declare that they have no competing interests.

## Authors’ contributions

Author Yi Ding, Xibiao Tang, Bin Wu, Zhuofei Xu, Lu Ping, Wugang Liu and Ruixuan Zhang performed the experiments. Author Weicheng Bei, Huanchun Chen and Chen Tan designed the experiments. Yi Ding, and Zhuofei Xu performed the analysis and interpretation of data. Yi Ding and Chen Tan wrote the manuscript. All the author approve submission of this manuscript to BMC Veterinary Research.

## Supplementary Material

Additional file 1 Table S1. Click here for file
